# Acetaldehyde via CGRP receptor and TRPA1 in Schwann cells mediates ethanol-evoked periorbital mechanical allodynia in mice: relevance for migraine

**DOI:** 10.1186/s12929-023-00922-6

**Published:** 2023-04-26

**Authors:** Lorenzo Landini, Daniel Souza Monteiro de Araujo, Martina Chieca, Gaetano De Siena, Elisa Bellantoni, Pierangelo Geppetti, Romina Nassini, Francesco De Logu

**Affiliations:** Department of Health Sciences, Clinical Pharmacology and Oncology Section, University of Florence, 50139 Florence, Italy

**Keywords:** Migraine, CGRP, Ethanol, Schwann cell, TRPA1, Oxidative stress

## Abstract

**Background:**

Ingestion of alcoholic beverages is a known trigger of migraine attacks. However, whether and how ethanol exerts its pro-migraine action remains poorly known. Ethanol stimulates the transient receptor potential vanilloid 1 (TRPV1) channel, and its dehydrogenized metabolite, acetaldehyde, is a known TRP ankyrin 1 (TRPA1) agonist.

**Methods:**

Periorbital mechanical allodynia following systemic ethanol and acetaldehyde was investigated in mice after TRPA1 and TRPV1 pharmacological antagonism and global genetic deletion. Mice with selective silencing of the receptor activated modifying protein 1 (RAMP1), a component of the calcitonin gene-related peptide (CGRP) receptor, in Schwann cells or TRPA1 in dorsal root ganglion (DRG) neurons or Schwann cells, were used after systemic ethanol and acetaldehyde.

**Results:**

We show in mice that intragastric ethanol administration evokes a sustained periorbital mechanical allodynia that is attenuated by systemic or local alcohol dehydrogenase inhibition, and TRPA1, but not TRPV1, global deletion, thus indicating the implication of acetaldehyde. Systemic (intraperitoneal) acetaldehyde administration also evokes periorbital mechanical allodynia. Importantly, periorbital mechanical allodynia by both ethanol and acetaldehyde is abrogated by pretreatment with the CGRP receptor antagonist, olcegepant, and a selective silencing of RAMP1 in Schwann cells. Periorbital mechanical allodynia by ethanol and acetaldehyde is also attenuated by cyclic AMP, protein kinase A, and nitric oxide inhibition and pretreatment with an antioxidant. Moreover, selective genetic silencing of TRPA1 in Schwann cells or DRG neurons attenuated periorbital mechanical allodynia by ethanol or acetaldehyde.

**Conclusions:**

Results suggest that, in mice, periorbital mechanical allodynia, a response that mimics cutaneous allodynia reported during migraine attacks, is elicited by ethanol via the systemic production of acetaldehyde that, by releasing CGRP, engages the CGRP receptor in Schwann cells. The ensuing cascade of intracellular events results in a Schwann cell TRPA1-dependent oxidative stress generation that eventually targets neuronal TRPA1 to signal allodynia from the periorbital area.

## Introduction

Along with a large number of endogenous and exogenous stimuli, including stress, fasting, and changes in sleep and hormone patterns, the drinking of alcoholic beverages has long been recognized as a common migraine trigger. Epidemiological studies [1–5] show that about one-third of migraine patients report alcohol as a trigger of their headaches. Ingestion of alcoholic beverages is reported to provoke two types of headaches: an acute type that starts within 3 h, and a delayed type (previously known as hangover headache) beginning several hours after alcohol ingestion. Both types resolve spontaneously within 72 h [6]. Although any individual may develop an alcohol-related headache, migraine sufferers appear more susceptible to the ability of alcoholic beverages to trigger headache or even typical migraine attacks [3, 4, 6]. Red and white wines have been reported as the alcoholic beverages that most frequently trigger headache attacks [7–10]. Although additional wine components, including histamine, polyphenols, sulfites, and others, have been proposed to contribute to the pro-migraine effect of alcoholic beverages [4, 11, 12], ethanol remains the major culprit as a provoking agent of headache attacks. However, the cellular and molecular mechanisms that cause ethanol-induced headache remain unknown.

Elevated local ethanol concentrations have been reported to lower from 42 to 35 °C degrees the threshold temperature for activation of the transient receptor vanilloid 1 (TRPV1) channel in mice, and by this mechanism to evoke a TRPV1-dependent acute nociception [13]. However, both local and systemic ethanol has been reported to elicit in the mouse hind paw a delayed and sustained mechanical allodynia that was attenuated by blocking acetaldehyde (ACD) generation and pharmacological antagonism or genetic silencing of the TRP ankyrin 1 (TRPA1) channel in Schwann cells surrounding nociceptors [14].

More recently, the mechanism underlying pain-like responses that follow excitation of the TRPV1+ve dorsal root ganglion (DRG) neuron has been elucidated in mice [15]. Whereas the acute nociceptive response evoked by the prototypical TRPV1 agonist, capsaicin, was entirely dependent on the direct drug action on and the ensuing afferent discharge of the excited peripheral terminals of TRPV1+ve DRG neurons, the delayed and sustained periorbital mechanical allodynia (PMA) relied on the release of calcitonin gene-related peptide (CGRP) from terminals of peptidergic DRG neurons [15]. CGRP stimulates its receptor (calcitonin receptor-like receptor/receptor activity modifying protein-1, CLR/RAMP1) in Schwann cells that wrap the sensory nerve terminals, thus generating a complex cascade of intracellular mediators that engage TRPA1 in Schwann cells. This results in the endoneurial release of reactive oxygen species (ROS) that is sustained for hours by a feed-forward TRPA1/ROS pathway and finally targets the neuronal TRPA1 to signal PMA [15].

In the last 20 years, preclinical and clinical findings have revealed the crucial role of CGRP as the major mediator of migraine pain [16, 17]. Here, we hypothesized that ethanol and/or ACD provoke migraine attacks by releasing CGRP. Migraine attacks are characterized by various pain symptoms, including unilateral throbbing pain and facial cutaneous allodynia [18, 19]. Thus, we investigated the cellular and molecular mechanisms underlying PMA evoked by ethanol and the contribution of ACD in mice. We report that ethanol ingestion elicits the PMA that is due to ACD and the ensuing CGRP release by peripheral terminals of DRG neurons. CGRP activates CLR/RAMP1 in Schwann cells to promote a cascade of intracellular events that result in a nitric oxide (NO)-mediated engagement of Schwann cell TRPA1 that produces a persistent ROS release, which in turn sustains the prolonged neuronal TRPA1 activation to signal allodynia.

## Materials and methods

### Mice

Male mice were use throughout (25–30 g, 5–8 weeks). In several mouse pain models where mechanical allodynia was mediated by CGRP and/or TRPA1 [15, 20, 21], no gender differences were found. Thus, in accordance with the 3Rs guidelines to minimize the number of animals, only male mice were used. The following strains of mice were used: C57BL/6 J mice (Charles River, RRID:IMSR_JAX:000664); wild-type (*Trpa1*^+*/*+^) and TRPA1-deficient (*Trpa1*^*−/*−^; B6129P-Trpa1^tm1Kykw^/J; RRID:IMSR_JAX:006401, Jackson Laboratory) mice [22]; wild-type (*Trpv1*^+*/*+^) and TRPV1-deficient (*Trpv1*^*−/−*^; B6129X1-Trpv1^tm1Jul^/J, RRID:IMSR_JAX:003770, Jackson Laboratory) mice. Genetically modified mice were maintained as heterozygotes on a C57BL/6 J background. To generate mice in which the *Trpa1* and *Ramp1* genes were conditionally silenced in Schwann cells/oligodendrocytes, homozygous 129S-Trpa1^tm2Kykw^/J (*floxed TRPA1, Trpa1*^*fl/fl*^, RRID:IMSR_JAX:008649 Jackson Laboratory) and C57BL/6N-Ramp1^<tm1c(EUCOMM)Wtsi>/H^ (*floxed* Ramp1, *Ramp1*^*fl/fl*^ Stock No: EM:07401, MRC HARWELL Mary Lion Center) [23] were crossed with hemizygous B6.Cg-Tg(Plp1-Cre^ERT^)3Pop/J mice (*Plp1-Cre*^*ERT*^, RRID:IMSR_JAX:005975 Jackson Laboratory), expressing a tamoxifen-inducible Cre in myelinating cells (Plp1, proteolipid protein myelin 1) [24]. The progeny (*Plp1-Cre*^*ERT*^;*Trpa1*^*fl/fl*^ and *Ramp1-Cre*^*ERT*^;*Trpa1*^*fl/fl*^) were genotyped by PCR for *Trpa1, Ramp1*, and *Plp1-Cre*^*ERT*^. Mice negative for *Plp1-Cre*^*ERT*^ (*Plp1-Cre*^*ERT−*^;*Trpa1*^*fl/fl*^ and *Plp1-Cre*^*ERT−*^;*Ramp1*^*fl/fl*^) were used as control. Both positive and negative mice to *Cre*^*ERT*^ and homozygous for floxed *Trpa1* (*Plp1-Cre*^*ERT*+^*;Trpa1*^*fl/fl*^ and *Plp1-Cre*^*ERT−*^*;Trpa1*^*fl/fl*^, respectively) and floxed *Ramp1 (Plp1-Cre*^*ERT*+^*;Ramp1*^*fl/fl*^ and *Plp1-Cre*^*ERT−*^*;Ramp1*^*fl/fl*^) mice were treated with intraperitoneal (i.p.) 4-hydroxytamoxifen (4-OHT) (1 mg/100 μl in corn oil, once a day for 3 consecutive days). Treatments resulted in Cre-mediated ablation of *Trpa1* and *Ramp1* in PLP-expressing Schwann cells/oligodendrocytes. To selectively delete the *Trpa1* gene in primary sensory neurons, *Trpa1*^*fl*^*/*^*fl*^ mice were crossed with hemizygous *Advillin-Cre* mice *(Adv-Cre)* [24–26]. Both positive and negative mice to *Cre* and homozygous for floxed *Trpa1* (*Adv-Cre*^+^*;Trpa1*^*fl/fl*^ and *Adv-Cre*^*−*^*;Trpa1*^*fl/fl*^, respectively) were used.

The group size of n = 8 animals for behavioral experiments was determined by sample size estimation using G*Power (v3.1) [27] to detect size effect in a post-hoc test with type 1 and 2 error rates of 5 and 20%, respectively. Mice were allocated to vehicle or treatment groups using a randomization procedure (http://www.randomizer.org/). Investigators were blinded to the identities (genetic background) and treatments, which were revealed only after data collection. No animals were excluded from experiments. The behavioral studies followed the animal research reporting in vivo experiment (ARRIVE) guidelines [28]. Mice were housed in a temperature- and humidity-controlled *vivarium* (12 h dark/light cycle, free access to food and water, 5 animals per cage). At least 1 h before behavioral experiments, mice were acclimatized to the experiment room and behavior was evaluated between 9:00 am and 5:00 pm. All the procedures were conducted following the current guidelines for laboratory animal care and the ethical guidelines for investigations of experimental pain in conscious animals set by the International Association for the Study of Pain [29]. Animals were anesthetized with a mixture of ketamine and xylazine (90 mg/kg and 3 mg/kg, respectively, i.p.) and euthanized with inhaled CO_2_ plus 10–50% O_2;_ confirmation of death was achieved by a physical method of killing (decapitation) (AVMA Guidelines for the Euthanasia of Animals, 2020).

### Behavioral experiments

#### Treatment protocol

Ethanol (15%, 1, 2 and 4 mL/kg) was given by intragastric (i.g.) and acetaldehyde (ACD) (0.01, 0.1 and 1 mg/kg) by i.p. route of administration. Vehicle of i.g. ethanol or i.p. ACD was 0.9% NaCl. Drug treatments were performed by i.p., i.g., or local periorbital (10 ml/site, p.orb.) route of administration. Vehicle of the various drugs was 4% dimethyl sulfoxide (DMSO) and 4% Tween-80 in 0.9% NaCl for p.orb. or i.p. administration and 0.5% carboxymethylcellulose for i.g. administration. Capsazepine (4 mg/kg, i.p.), 4-methylpyrazole (4-Mp, 50 mg/kg, i.g.) or vehicle were administered 30 min before ethanol (15%, 4 ml/kg, i.g.). A967079, [(1E,3E)-1-(4-Fluorophenyl)-2-methyl-1-penten-3-one oxime], phenyl-N-tert-butyl nitrone (PBN) (100 mg/kg, i.p.), or vehicle were administered 2 h after ethanol (15%, 4 ml/kg, i.g.) or ACD (0.1 mg/kg, i.p.). 4-Mp (100 nmol), A967079 (300 nmol), PBN (670 nmol), olcegepant (1 nmol), SQ-22536 (25 nmol), H89 (45 nmol), L-NAME (1 mmol) cPTIO (200 nmol), or vehicle were administered locally (p.orb.) 30 min before or 2 h after ethanol (15%, 4 ml/kg, i.g.) or ACD (0.1 mg/kg, i.p.). If not otherwise indicated, reagents were obtained from Merck Life Science (Milan, Italy).

#### Periorbital mechanical allodynia

Facial cutaneous allodynia is one component of the pain symptoms of migraine attack [30], and 70% of migraineurs experience cephalic allodynia [18]. Thus, the measurement of mechanical thresholds in the periorbital area is considered a reliable method to investigate the mechanisms underlying headache and migraine pain [31]. PMA was assessed using the up-down paradigm [32, 33]. Briefly, mice were placed in a restraint apparatus designed for the evaluation of periorbital mechanical thresholds [34]. One day before the first behavioral observation, mice were habituated to the apparatus. PMA was evaluated in the periorbital region over the rostral portion of the eye (i.e., the area of the periorbital region facing the sphenoidal rostrum) [35] before (basal threshold) and after (0.5, 1, 2, 4, 6, 8, 24 h) treatments. On the day of the experiment, after 20 min of adaptation inside the chamber, a series of 7 von Frey filaments in logarithmic increments of force (0.02, 0.04, 0.07, 0.16, 0.4, 0.6 and 1.0 g) were applied to the periorbital area perpendicular to the skin, with sufficient force to cause slight buckling, and held for approximately 5 s to elicit a positive response. Mechanical stimuli were applied homolaterally outside the periorbital area at a distance of 6–8 mm from the site where compounds were injected. The response was considered positive by the following criteria: mouse vigorously stroked its face with the forepaw, head withdrawal from the stimulus, or head shaking. Mechanical stimulation started with the 0.16 g filament. Absence of response after 5 s led to the use of a filament with increased force, whereas a positive response led to the use of a weaker (i.e., lighter) filament. Six measurements were collected for each mouse or until four consecutive positive or negative responses occurred. The 50% mechanical withdrawal threshold (expressed in g) was then calculated from these scores by using a δ value of 0.205, previously determined [35].

#### Rota-rod test

Locomotor function, balance, and sedation of mice was assessed after drug administration. The animals were trained on a rotarod apparatus (Ugo Basile) 24 h before the test. The day of the experiment, each mouse was individually placed on the apparatus, which accelerated from 4 to 40 rpm over the trial time of 300 s. Latency to fall was evaluated and recorded for three trials.

### Acetaldehyde assay

The infraorbital trunk of the trigeminal nerve was obtained from euthanized mice before and 15, 30, 60 and 180 min after ethanol (15%, 4 ml/kg, i.g.) administration. Tissues were homogenized in phosphate buffer saline (PBS, 0.1 M) by using a tissue homogenizer (Qiagen SpA, Milan, Italy) for 3 min, centrifuged 10,000 rpm for 10 min, and supernatants collected. Acetaldehyde content (expressed as nmol/mg of protein) was determined by using a colorimetric assay (Megazyme, Ireland) according to the manufacturer’s protocol.

### Immunofluorescence

Mice were anesthetized, transcardially perfused with PBS (phosphate buffer saline) followed by 4% paraformaldehyde, and the infraorbital trunk of the trigeminal nerve was collected, post-fixed for 24 h, transferred to 30% sucrose overnight, frozen, and cryosectioned at 10 μm. The slides were then incubated with the 4-hydroxy-nonenal (4-HNE) primary antibody (#ab48506, HNEJ-2, mouse monoclonal, 1:25, Abcam) and S100 (#ab196175, recombinant Alexa Fluor 647 anti-S100, rabbit monoclonal, 1:200, Abcam) diluted in fresh blocking solution (PBS, pH 7.4, 5% normal goat serum, NGS) 1 h at room temperature (RT), followed by a fluorescent polyclonal secondary antibody Alexa Fluor 488 (1:600; Invitrogen) 2 h at RT. Slides were then coverslipped with mounting medium with 4′,6-diamidino-2-phenylindole (DAPI, #ab228549, Abcam). Fluorescence images were obtained using an AxioImager 2 microscope (Carl Zeiss). The fluorescence intensity of 4-HNE staining was evaluated by the image processing module of ZEN Pro (Carl Zeiss).

### H_2_O_2_ assay

The infraorbital trunk of the trigeminal nerve was obtained from euthanized mice before and 1, 3, 6 and 8 h after ethanol (15%, 4 ml/kg, i.g.) or acetaldehyde (0.1 mg/kg, i.p.) administration. The H_2_O_2_ content was determined by using the Amplex Red® assay (Invitrogen, Milan, Italy). Briefly, tissue was rapidly removed and placed into modified Krebs/HEPES buffer (composition in mmol/l: 99.01 NaCl, 4.69 KCl, 2.50 CaCl_2_, 1.20 MgSO_4_, 1.03 KH_2_PO_4_, 25.0 NaHCO_3_, 20.0 Na-HEPES, and 5.6 glucose [pH 7.4]). Samples were minced and incubated with Amplex red (100 µM) and HRP (1 U/ml) (1 h, 37 °C) in modified Krebs/HEPES buffer protected from light [24]. Fluorescence excitation and emission were at 540 and 590 nm, respectively. H_2_O_2_ production was calculated using H_2_O_2_ standard and expressed as µmol/l of mg of dry tissue.

### Statistical analysis

Results are expressed as mean ± standard error of the mean (SEM). For multiple comparisons, a one-way analysis of variance (ANOVA) followed by the post-hoc Bonferroni’s test was used. For behavioral experiments with repeated measures, the two-way mixed model ANOVA followed by the post-hoc Bonferroni’s test was used. Statistical analyses were performed on raw data using Graph Pad Prism 8 (GraphPad Software Inc.). P values less than 0.05 (P < 0.05) were considered significant. Statistical tests used and the sample size for each analysis are listed in the figure legends.

## Results

### Periorbital mechanical allodynia by ethanol is mediated by acetaldehyde

**Fig. 1:**
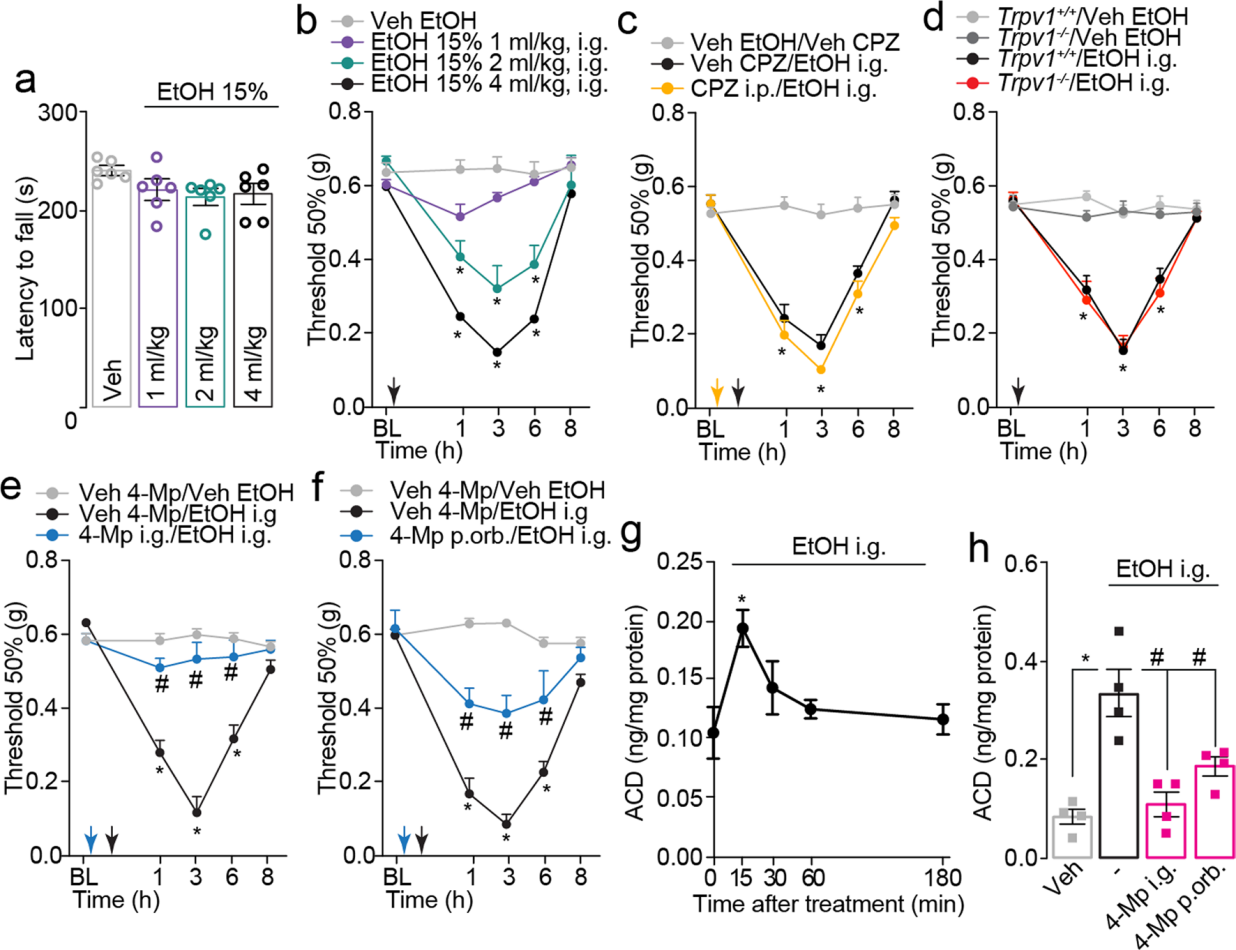
Acetaldehyde sustains ethanol (EtOH)-evoked periorbital mechanical allodynia (PMA). **a** Dose-dependent motor coordination and **b** dose- and time-dependent PMA evoked by EtOH (intragastric, i.g.) or vehicle (veh, 0.9% NaCl) in C57BL/6J mice. **c** PMA evoked by EtOH (15%, 4 ml/kg, i.g.) or veh in C57BL/J mice pretreated (30 min before) with capsazepine (CPZ, 4 mg/kg, i.p.) or veh. **d** PMA evoked by EtOH (15%, 4 ml/kg, i.g.) or veh in *Trpv1*^+*/*+^ and *Trpv1*^*−/−*^ mice. **e**, **f** PMA evoked by EtOH (15%, 4 ml/kg, i.g.) or veh in C57BL/6J mice pretreated (30 min before) with 4-methylpyrazole (4-M, 50 mg/kg, i.g. or 100 nmol, periorbital, p.orb.), or veh (n = 8 mice for behavioral experiments). **g** Acetaldehyde (ACD) levels in the infraorbital trunk of the trigeminal nerve of C57BL/6J mice receiving EtOH (15%, 4 ml/kg, i.g.) (n = 4 mice for each condition). **h** ACD levels in the infraorbital trunk of the trigeminal nerve of C57BL/6J mice 3 h after EtOH (15%, 4 ml/kg, i.g.) or veh and pretreated (30 min before) with 4-M (50 mg/kg, i.g. or 100 nmol, p.orb.) (n = 4 mice for each condition). Dash (-) is the combination of veh. *P < 0.05 vs. veh, time 0; ^#^P < 0.05 vs. 4-Mp-EtOH, one and two-way ANOVA with Bonferroni post-hoc correction

To mimic the pain symptoms of migraine attacks produced by ingestion of alcoholic beverages, graded doses of ethanol (1–4 mL/kg of ethanol 15% in NaCl) were given to mice by the intragastric (i.g.) route of administration. Even at the highest dose (4 mL/kg) of i.g., ethanol mice did not show measurable acute spontaneous nociceptive behavior or impairment of motor coordination (Fig. 1a). However, ethanol evoked a dose-related, delayed and sustained PMA (~ 6 h) (Fig. 1b) that was unaffected by pretreatment with the TRPV1 antagonist, capsazepine, or in mice with global TRPV1 deletion, (Fig. 1c, d). Because this type of pain-related response was independent from the activation of the channel that is known to be excited by ethanol [13], we explored the implication of other pathways.

The direct ethanol dehydrogenated metabolite, ACD, has previously been found to induce a delayed mechanical allodynia in the mouse hind paw [14]. Here, we show that systemic (i.g.) or local (p.orb.) pretreatment with the selective ADH inhibitor, 4-Mp, produced a total or partial reduction of ethanol-evoked PMA, respectively (Fig. 1e, f). Increased ACD levels were found in the infraorbital trunk of the trigeminal nerve of C57BL/6J mice after i.g. ethanol (Fig. 1g). Ingested alcohol is mainly metabolized to ACD by hepatic ADH [36], although extrahepatic ADH may also contribute [37, 38]. Following i.g. ethanol, increased ACD levels in the infraorbital trunk of the trigeminal nerve were totally or partially reduced by pre-treatment with i.g. or p.orb. 4-Mp, respectively (Fig. 1h). As p.orb. 4-Mp elicited a partial inhibition of ACD generation and ethanol-evoked PMA, it is possible that ACD of both hepatic and local origin contribute to ethanol-evoked PMA.

### Acetaldehyde elicits PMA by engaging CGRP receptor and TRPA1 in Schwann cells

**Fig. 2:**
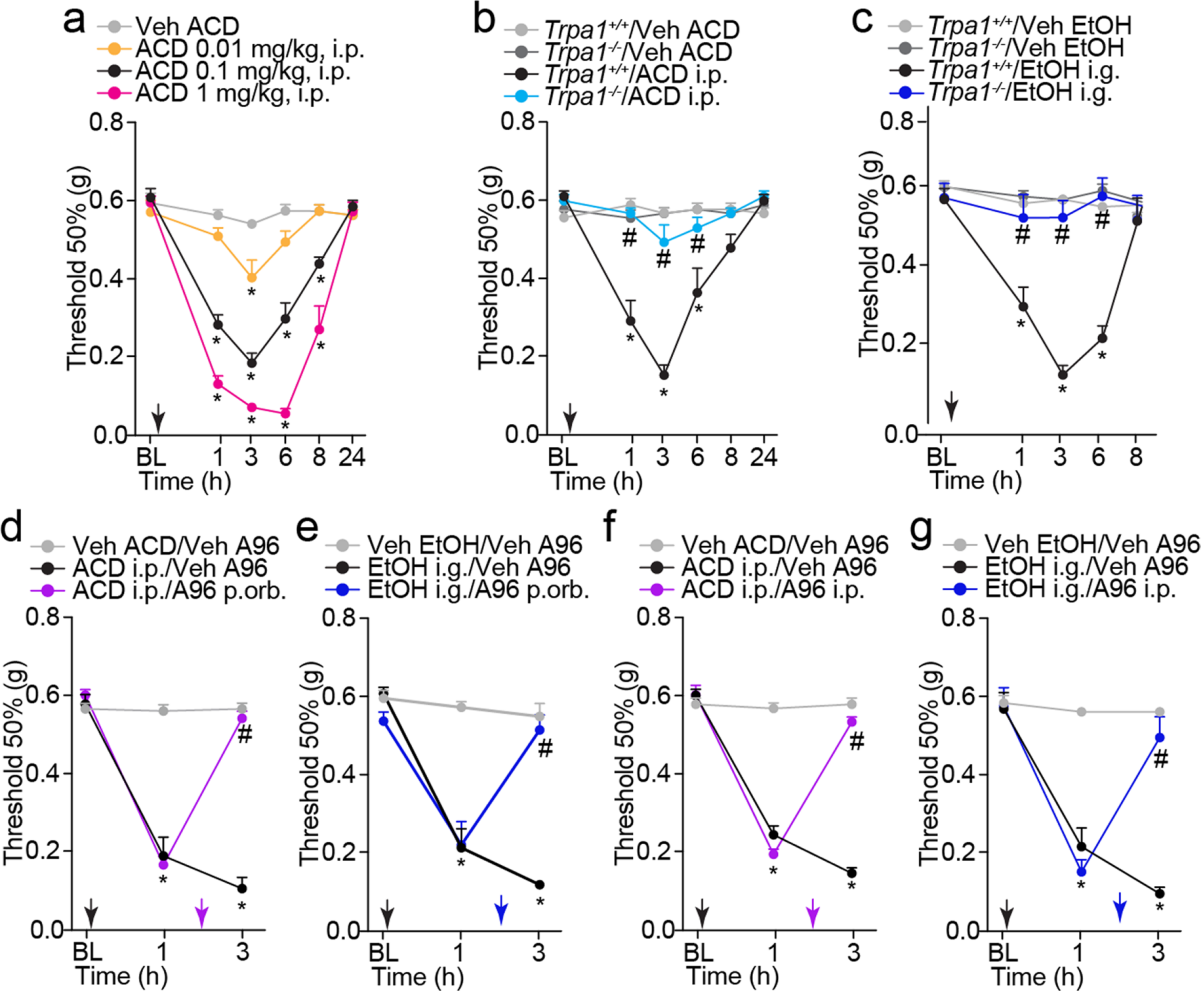
TRPA1 mediates periorbital mechanical allodynia (PMA) evoked by ethanol (EtOH) and acetaldehyde (ACD). **a** Dose- and time-dependent PMA evoked by ACD (i.p.) or vehicle (veh, 0.9% NaCl) in C57BL/6 J mice. **b**, **c** PMA evoked by ACD (0.1 mg/kg, i.p.), EtOH (15%, 4 ml/kg, i.g.), or veh in *Trpa1*^+*/*+^ and *Trpa1*^*−/−*^ mice. **d**, **e** PMA evoked by ACD (0.1 mg/kg, i.p.), EtOH (15%, 4 ml/kg, i.g.), or veh in C57BL/6J mice treated (2 h after) with A967079 (A96 300 nmol, periorbital, p.orb.) or veh. (f, g) PMA evoked by ACD (0.1 mg/kg, i.p.), EtOH (15%, 4 ml/kg, i.g.), or veh in C57BL/6J mice treated (2 h after) with A96 (100 mg/kg, i.p.) or veh. (n = 8 mice). *P < 0.05 vs. veh; ^#^P < 0.05 vs. *Trpa1*^+*/*+^-EtOH, *Trpa1*^+*/*+^-ACD, veh A96-EtOH, ACD, two-way ANOVA with Bonferroni post-hoc correction

**Fig. 3:**
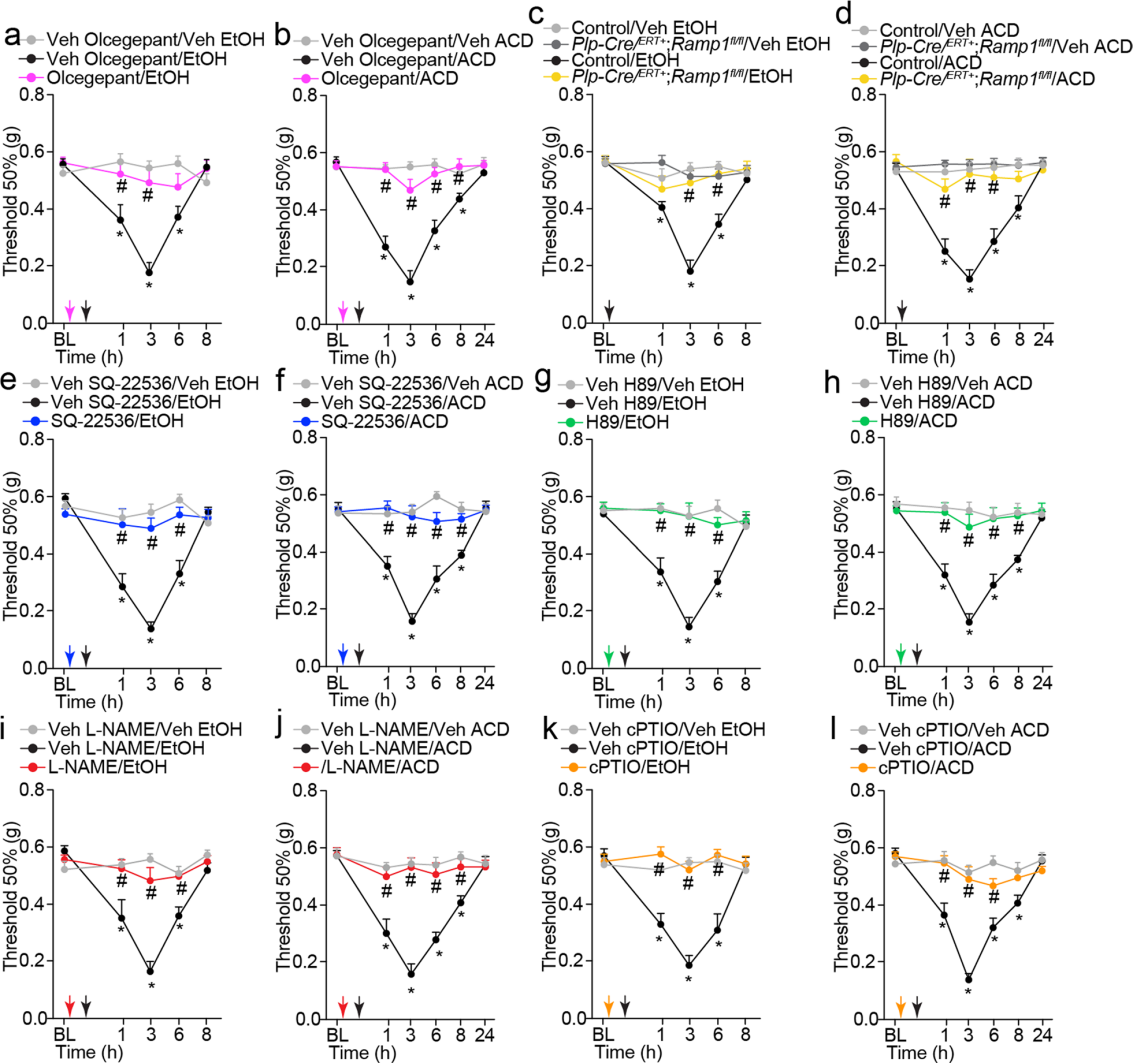
Schwann cell CGRP receptor activation mediates periorbital mechanical allodynia (PMA) evoked by ethanol (EtOH) and acetaldehyde (ACD). **a**, **b** Time-dependent PMA evoked by EtOH (15%, 4 ml/kg, i.g.), ACD (0.1 mg/kg, i.p.), or vehicle (veh, 0.9% NaCl) in C57BL/6J mice treated (30 min before) with olcegepant (1 nmol, periorbital, p.orb.) or veh. **c**, **d** PMA evoked by EtOH (15%, 4 ml/kg, i.g.), acetaldehyde (ACD, 0.1 mg/kg, i.p.), or veh in *Plp1-Cre*^*ERT*+^*;Ramp1*^*fl/fl*^ and *Plp1-Cre*^*ERT−*^*;Ramp1*^*fl/fl*^ (control) mice. PMA evoked by EtOH (15%, 4 ml/kg, i.g.), acetaldehyde (ACD, 0.1 mg/kg, i.p.), or vehicle (veh, 0.9% NaCl) in C57BL/6J mice treated (30 min before) with **e**, **f** SQ-22536 (25 nmol, p.orb.), **g**, **h** H89 (45 nmol, p.orb.), **i**, **j** L-NAME (1 mmol, p.orb.), **k**, **l** cPTIO (200 nmol, p.orb.), or veh. (n = 8 mice). *P < 0.05 vs. veh; ^#^P < 0.05 vs. veh olcegepant, SQ-22536, H89, L-NAME, cPTIO, control -EtOH, two-way ANOVA with Bonferroni post-hoc correction

Systemic (i.p.) injection of ACD caused a dose-dependent, delayed, and prolonged PMA (~ 6 h) (Fig. 2a). PMA by both ACD (i.p.) and ethanol (i.g.) was absent in mice with TRPA1 global deletion (Fig. 2b, c) and reversed by local (p.orb.) or systemic (i.p.) (Fig. 2d–g) post-treatment (2 h after ACD or ethanol) with the TRPA1 antagonist, A967079. TRPA1+ve DRG neurons, which account for ~ 30% of TRPV1+ve neurons [39], contain neuropeptides, including CGRP, that can be released by exogenous or endogenous channel agonists [40–42]. Notably, PMA evoked by ethanol (i.g.) or ACD (i.p.) was prevented by pretreatment with the CGRP receptor antagonist, olcegepant (Fig. 3a, b). Thus, CGRP, responsible for PMA evoked by i.g. ethanol, is released by its rapid systemic and/or local conversion into the TRPA1 agonist, ACD.

Recently, we reported that exogenous or endogenous (released by capsaicin) CGRP provokes PMA via Schwann cell CLR/RAMP1 activation [15]. PMA evoked by ethanol (i.g.) or ACD (i.p.) was markedly attenuated in mice with Schwann cell selective silencing of the RAMP1 subunit of the CGRP receptor (*Plp-Cre*^*ERT*+^*;Ramp1*^*fl/fl*^) but not in control (*Plp-Cre*^*ERT−*^*;Ramp1*^*fl/fl*^) mice (Fig. 3 c,d). Schwann cell CLR/RAMP1 activation results in a series of intracellular events that comprise cAMP release, protein kinase A (PKA) activation, endothelial nitric oxide synthase (eNOS) phosphorylation, and nitric oxide (NO) release [15]. Here, the intracellular pathway responsible for capsaicin-evoked PMA was recapitulated by PMA evoked by ethanol (i.g.) or ACD (i.p.). Pre-treatment with the adenyl cyclase inhibitor, SQ-22536 (Fig. 3e, f), the PKA inhibitor, H89 (Fig. 3g, h), the NOS inhibitor, L-NAME (Fig. 3i, j), or the NO scavenger, cPTIO (Fig. 3k, l), abrogated PMA induced by ethanol (i.g.) or ACD (i.p.).

### NO induces a Schwann cell TRPA1-dependent sustained ROS release that targets the DRG neuron TRPA1 to signal allodynia

**Fig. 4:**
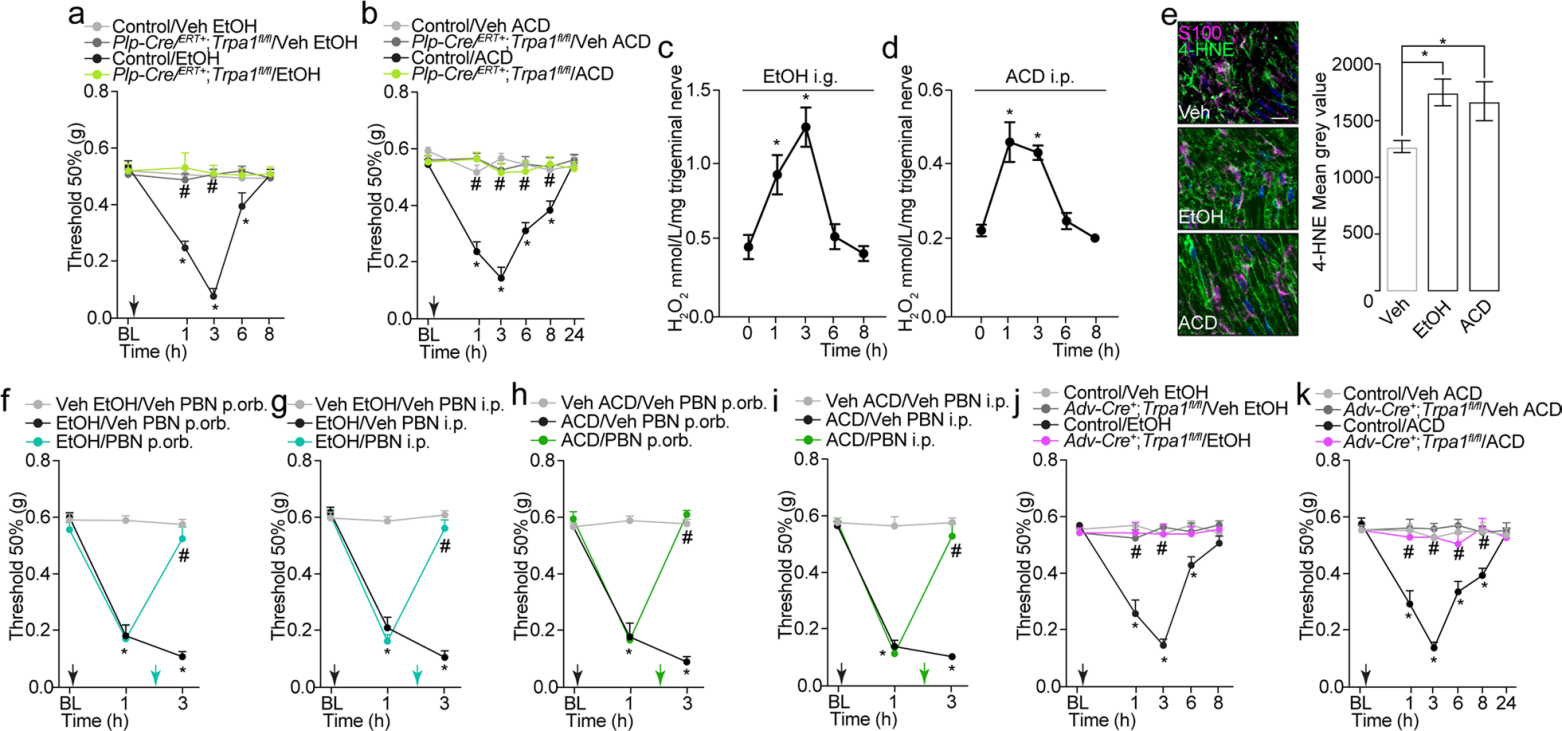
Schwann cell TRPA1 receptor activation mediates periorbital mechanical allodynia (PMA) evoked by ethanol (EtOH) and acetaldehyde (ACD). **a**, **b** Time-dependent PMA evoked by EtOH (15%, 4 ml/kg, i.g.), ACD (0.1 mg/kg, i.p.), or vehicle (veh, 0.9% NaCl) in *Plp1-Cre*^*ERT*+^*;Trpa1*^*fl/fl*^ and *Plp1-Cre*^*ERT−*^*; Trpa1*^*fl/fl*^ (control) mice. **c**, **d** H_2_O_2_ levels in the infraorbital trunk of the trigeminal nerve of C57BL/6J mice receiving EtOH (15%, 4 ml/kg, i.g.), ACD (0.1 mg/kg, i.p.) or veh. **e** Representative photomicrographs and mean fluorescence intensity of 4-hydroxy-nonenal (4-HNE) staining in the infraorbital trunk of the trigeminal nerve of C57BL/6J mice receiving EtOH (15%, 4 ml/kg, i.g.), ACD (0.1 mg/kg, i.p.), or veh (0.9% NaCl) (scale bar: 10 µm) (n = 4 mice for each condition). PMA evoked by **f**, **g** EtOH (15%, 4 ml/kg, i.g.), **h**, **i** ACD (0.1 mg/kg, i.p.) or veh in C57BL/6J mice treated (2 h after) with PBN (100 mg/kg, i.p. or 670 nmol, periorbital, p.orb.) or veh. **j**, **k** PMA evoked by EtOH (15%, 4 ml/kg, i.g.), ACD (0.1 mg/kg, i.p.), or veh in *Adv-Cre*^+^*;Trpa1*^*fl/fl*^ and *Adv-Cre*^*−*^*;Trpa1*^*fl/fl*^ (control) mice. (n = 8 mice). *P < 0.05 vs. veh, time 0; ^#^P < 0.05 vs. control -EtOH, ACD, *Adv-Cre*^*−*^*;Trpa1*^*fl/fl*^ -EtOH, ACD, veh PBN-EtOh, ACD

NO, most likely via its derivatives, including nitroxyl (HNO) [43], targets TRPA1 through reversible covalent modification (nitrosylation) of cysteine residues [44]. *In vitro* stimulation of human Schwann cells with CGRP induced a TRPA1-dependent calcium response, which, being inhibited by L-NAME and cPTIO, implicated a role of NO or its derivatives [15]. PMA evoked by both ethanol (i.g.) and ACD (i.p.) was prevented in mice with selective silencing of TRPA1 (*Plp-Cre*^*ERT*+^*;Trpa1*^*fl/fl*^) in Schwann cells (Fig. 4a,b). Schwann cell TRPA1 activation was reported to produce a NADPH oxidase synthase 1 (NOX1)-dependent generation of H_2_O_2_ [15]_._ Both ethanol (i.g.) and ACD (i.p.) induced a sustained (~ 3 h) accumulation of H_2_O_2_ in the infraorbital trunk of the trigeminal nerve of C57BL/6J mice (Fig. 4c,d). In addition, the presence of 4-HNE, an end-product of oxidative stress [45], was explored in sections of the infraorbital trunk of the trigeminal nerve of C57BL/6J mice following ethanol (i.g.) or ACD (i.p.) administration. Three h after ethanol or ACD administration, an increased 4-HNE staining was observed throughout the infraorbital trunk of the trigeminal nerve, including Schwann cells, as indicated by its colocalization with the specific Schwann cell marker, S100 (Fig. 4e). Posttreatment (p.orb. or i.p., 2 h after ACD or ethanol) of C57BL/6 J mice with the antioxidant spin trap agent, PBN, reversed PMA induced by ethanol (i.g.) (Fig. 4f, g) or ACD (i.p.) (Fig. 4h, i). PMA induced by ethanol (i.g.) or ACD (i.p.) was abolished in mice with selective silencing of TRPA1 in primary sensory neurons (*Adv-Cre*^+^*;Trpa1*^*fl/fl*^) (Fig. 4j, k), thus supporting the view that prolonged ROS generation targets neuronal TRPA1 to signal PMA associated with ethanol ingestion.

## Discussion

Among dietary causes, alcohol consumption has been reported as one of the most frequent triggers for migraine attacks in susceptible individuals [3]. To mimic the ingestion of alcoholic beverages, ethanol was given to mice by intragastric gavage. Although it is known that ethanol ingestion may alter motor coordination in mice [46], the present doses of ethanol given by gavage did not affect motor coordination in C57BL/6J mice. Furthermore, no measurable acute nociceptive response was observed after ethanol intragastric administration. However, as we previously found in the mouse hind paw [14], i.g. ethanol provoked a prolonged PMA that was resistant to TRPV1 pharmacological antagonism or global genetic deletion.

Thus, although ethanol has been recognized to gate TRPV1 by lowering its threshold temperature for activation [13], the most parsimonious explanation for the present results is that the relatively high local concentrations of ethanol necessary to evoke the TRPV1-mediated acute nociception are not achieved by the i.g. doses of ethanol administered. Notably, the delayed and prolonged occurrence of PMA in mice that received i.g. ethanol suggests this model as a satisfactory method to reproduce the delayed headache that, in migraine patients, initiates within 3 or more hours and may last up to 72 h from the ingestion of alcoholic beverages [6]. Thus, the delayed onset and the sustained (hours) PMA produced in mice by i.g. ethanol and the time course of migraine headache following ethanol ingestion in patients strengthen the translational value of the present findings. The observation that ethanol exhibits similar pharmacokinetics in humans and mice [47, 48] further supports this analogy.

Although ethanol may target TRPV1, PMA that follows ethanol administration in mice is independent of TRPV1. This apparent contradiction may be explained by considering that ethanol undergoes rapid metabolism to ACD prevalently, but not exclusively, in the liver [36, 38]. Our observation that the ADH inhibitor, 4-Mp, prevented both the increase in ACD levels and PMA caused by i.g. ethanol robustly supports the view that ACD is the main mediator of alcohol-evoked mechanical allodynia. In addition, the contribution of locally (p.orb.) produced ACD is supported by the finding that local ADH inhibition by (p.orb.) 4-Mp reduced PMA by systemic (i.g.) ethanol. Importantly, 4-Mp data underline that ACD is necessary and sufficient to mediate the delayed allodynia in the periorbital area. The observations that disulfiram, which blocks the transformation of ACD into acetic acid, in combination with alcohol intake, but not per se, increases the possibility of developing headaches [6], and that when Asians, with loss of function ALDH2 mutations, consume alcohol, acetaldehyde accumulating in the blood can lead to physical discomfort and various symptoms, including headache [49], further strengthen the role of ACD as a headache-provoking agent.

The known activity of ACD to gate TRPA1 [14, 50] indicated this channel as the proalgesic mechanism activated by the ethanol metabolite. The hypothesis was further corroborated by pharmacological antagonism and genetic deletion of TRPA1. Reduction in i.g. ACD-evoked PMA by TRPA1 pharmacological antagonism or in mice with global TRPA1 deletion is consistent with our hypothesis that ACD gates TRPA1 and plays a major role in ethanol-induced migraine-related PMA.

However, the major finding of the present study is the identification of the role of CGRP in ethanol-evoked PMA. Capsaicin and ethanol are known to release the pro-migraine neuropeptide, CGRP [16, 17], from terminals of TRPV1+ve and TRPA1+ve DRG neurons [51–55]. As recently reported for capsaicin [15], here, results based on pharmacological CGRP receptor antagonism with olcegepant indicate the critical role of the neuropeptide in the PMA elicited by ethanol ingestion in mice. Notably, experiments in mice with selective silencing of RAMP1 in Schwann cells revealed the crucial role of CLR/RAMP1 targeting by CGRP in these peripheral glial cells. The results raise the question of the subtype of the Schwann cells implicated in the CGRP-mediated allodynia. Unmyelinated C-fiber nociceptors are the major source of sensory neuropeptides, including CGRP, while a minor contribution is given by thinly myelinated Aδ-fiber nociceptors [56]. Thus, the most parsimonious hypothesis is that CGRP is released mainly by ethanol derived ACD from the varicosities of terminals of unmyelinated C-fiber nociceptors. On this basis, and hypothesizing that CGRP-evoked allodynia is produced within the first cell that CGRP encounters upon its release from terminal varicosities, we propose that CGRP targets CLR/RAMP1 expressed by the unmyelinated Remak subtype of Schwann cells that surround C-fiber nociceptors.

**Fig. 5:**
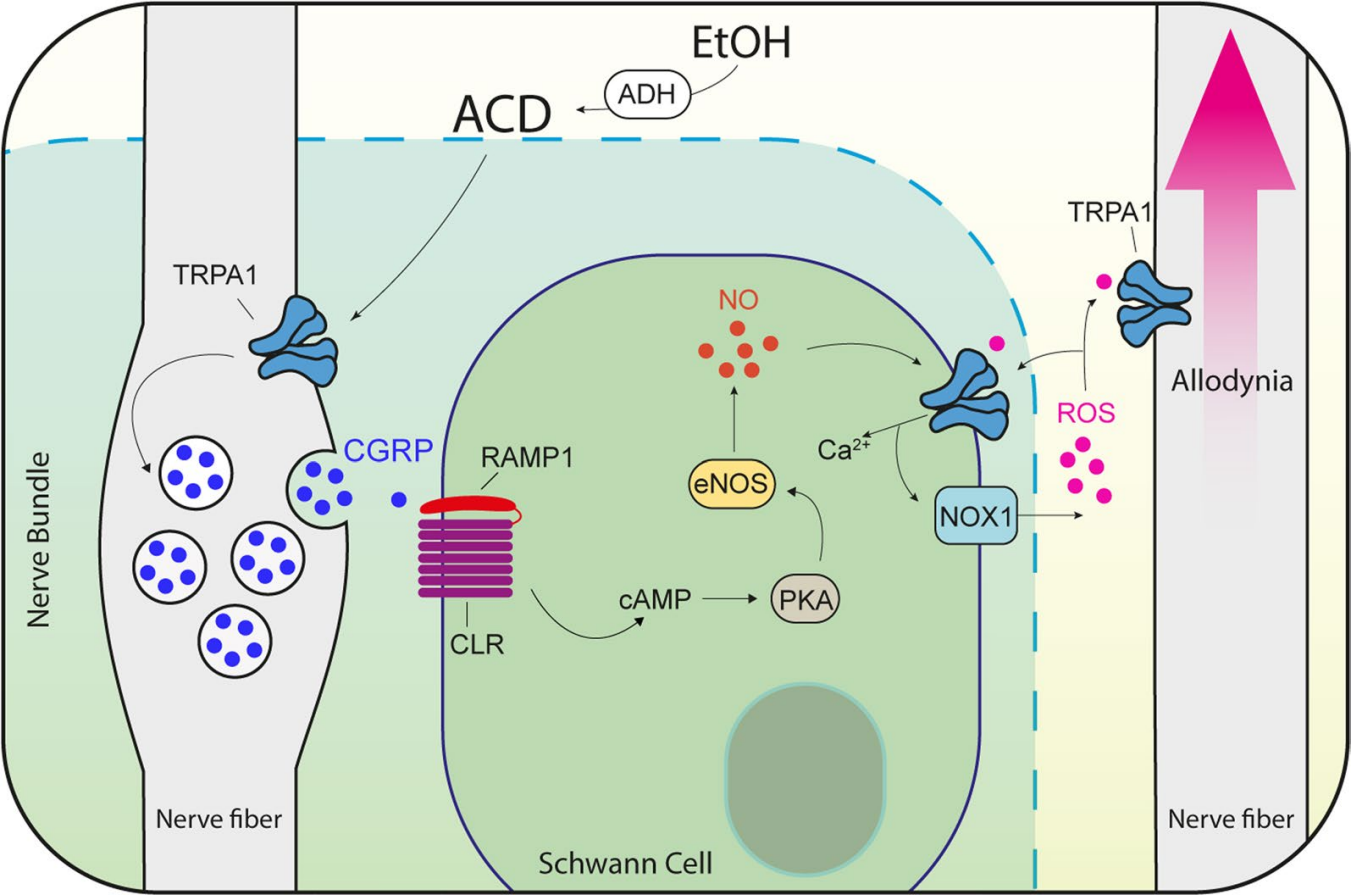
Schematic representation of the pathway that signals cutaneous allodynia elicited by the ethanol (EtOH) metabolite, acetaldehyde (ACD). ACD, by targeting TRPA1, releases CGRP from trigeminal nerve terminals that activate CLR/RAMP1 on Schwann cells. CLR/RAMP1 activation elicits a transient cAMP-mediated protein kinase A (PKA) phosphorylation of endothelial nitric oxide synthase (eNOS). The ensuing nitric oxide (NO) release targets the oxidant-sensitive channel, TRPA1, in Schwann cells, which sustains and amplifies reactive oxygen species (ROS) generation. The persistent oxidative stress signals mechanical allodynia via TRPA1 on adjacent nerve fibers. ADH alcohol dehydrogenase

The cascade of intracellular mediators implicated in PMA in the present mouse model evoked by ethanol recalls the pathway previously identified in cultured human and mouse Schwann cells and in mice in vivo exposed to CGRP [15]. This pathway comprises early and transient increases in cyclic AMP and NO and a sustained TRPA1-dependent feed-forward mechanism that results in a prolonged ROS generation. In fact, the increased hydrogen peroxide levels in the infraorbital trunk of the trigeminal nerve and the ability of the antioxidant agent, PBN, and the TRPA1 antagonist, A967079, to attenuate PMA not only when given prior to, but also after, ethanol or ACD underlines the prolonged role of oxidative stress generation and TRPA1 engagement in sustaining the protracted ethanol-evoked PMA. Finally, the key role of Schwann cell and DRG neuron TRPA1, originally advanced in the pathway responsible for capsaicin-evoked PMA [15], has been reproduced here when PMA evoked by i.g. ethanol and i.p. ACD was investigated. In fact, the present functional experiments point to TRPA1 expressed by C-fiber nociceptor as the target of ACD to release CGRP that promotes ethanol-evoked PMA. However, the present data suggest that TRPA1 contributions to ethanol-evoked PMA are multiple, and not solely confined to the channel expressed by the C-fiber nociceptor, but imply two additional cellular sites where TRPA1 may contribute to the pain-like response. In fact, genetic silencing of TRPA1 in Schwann cells implicates the channel expressed by these glial cells as the target of NO, or other more reactive NO derivatives, including HNO [43], which results in the prolonged ROS generation. Finally, the Schwann cell TRPA1/ROS pathway, in a feed-forward manner, amplifies the oxidative stress that persistently signals mechanical allodynia via the neuronal TRPA1. Although this issue cannot be conclusively determined, indirect evidence [57] suggests that the neuronal TRPA1 responsible for ethanol-evoked PMA may be that expressed by non-peptidergic Aδ-fiber DRG neurons (Fig. 5).

In a previous paper [14], we reported that subcutaneous injection of ACD in the mouse hind paw elicits both acute and transient (10 min) nociception and delayed and prolonged (6 h) mechanical allodynia. However, in agreement with the present findings, systemic (intraperitoneal) ACD only elicited delayed allodynia [14]. The difference may be due to the local amount of ACD that, following subcutaneous administration, achieves levels high enough to enable the targeting the TRPA1 expressed by both peptidergic nerve terminals that release CGRP and terminals that signal allodynia. Alternatively, as we reported [14] that Schwann cells contribute to the local transformation of ethanol into ACD, it is possible that this metabolic pathway occurs in Schwann cells that surround peptidergic nerve fibers, but not the nerve fibers that signal allodynia. However, both hypotheses remain highly speculative, and further studies are needed to answer this important issue.

## Conclusion

The present study reveals that, in mice, ethanol ingestion elicits PMA indirectly via its metabolite, ACD, that, by targeting TRPA1 in peptidergic trigeminal nerve fibers, releases CGRP. CGRP engages its receptor in Schwann cells, thus promoting a series of intracellular events that results in a sustained amplification of oxidative stress mediated by Schwann cell TRPA1. ROS generated by this pathway eventually targets neuronal TRPA1 to signal allodynia from the periorbital area. The mechanisms reported in the present study further support the role of CGRP and Schwann cells in generating cutaneous mechanical allodynia, a frequently reported symptom during migraine attacks induced by dietary migraine provoking agents, such as alcoholic beverages.

## Data Availability

Data generated during the current study are available from the corresponding author on reasonable request.
